# Antibiotic Residues in Meat and Animal Feed and Their Association with Antimicrobial Resistance: Evidence from the Kostanay Region, Kazakhstan

**DOI:** 10.3390/foods15112042

**Published:** 2026-06-05

**Authors:** Pavel Shevchenko, Zhanaidar Bermukhametov, Albina Gabitova, Alma Dossova, Bakhit Baimenov, Aliya Yskak, Gulnaz Yermoldina, Oxana Tomaruk, Raushan Rychshanova

**Affiliations:** Research Institute of Innovative Technologies, NPLC “Akhmet Baitursynuly Kostanay Regional University”, 110000 Kostanay, Kazakhstan; pavel87011339688@gmail.com (P.S.); zhanaidar007@gmail.com (Z.B.); bibishka03@gmail.com (A.G.); dossova.alma95555@gmail.com (A.D.); bahytbajmenov@gmail.com (B.B.); yskak_aliya@ksu.edu.kz (A.Y.); yermoldina.g@ksu.edu.kz (G.Y.)

**Keywords:** antibiotic residues, animal feed, meat, antimicrobial resistance, food chain, food safety, cross-contamination

## Abstract

The widespread use of antibacterial agents in livestock production is associated with antibiotic residues in animal products and feed, posing a potential threat to food safety and contributing to antimicrobial resistance. The aim of this study was to assess contamination levels of beef, pork, and feed with antibiotic residues in the Kostanay region. The results demonstrated the presence of antibiotic residues in beef, pork, and feed, while all detected concentrations remained below established maximum residue limits. Despite compliance with regulatory standards, residues were detected frequently, and their levels differed significantly depending on feed type (*p* < 0.001). Microbiological analysis confirmed the presence of *Escherichia coli* and *Staphylococcus aureus* in meat samples, with isolates exhibiting moderate to high antimicrobial resistance, particularly in pork. The highest resistance was observed to tetracycline, streptomycin, and thiamphenicol. The obtained data indicate that even trace antibiotic concentrations entering the “feed–animal–product” chain may be associated with selective pressure on microbiota and the circulation of resistant microorganisms. The results highlight the need for comprehensive monitoring of feed and animal products to assess and mitigate antimicrobial resistance spread within the food chain.

## 1. Introduction

The widespread use of veterinary medicinal products is an integral part of modern animal husbandry, driven by the need for the prevention and treatment of infectious diseases. Antibacterial drugs from various groups, including β-lactams, tetracyclines, aminoglycosides, sulfonamides, macrolides, and fluoroquinolones, are among the most commonly used. However, the intensive and often uncontrolled use of antibiotics leads to the formation of residual amounts of these substances in food products of animal origin, which is considered a major food safety concern [1,2,3,4].

Residual amounts of antibiotics are most frequently detected in meat and milk. Analysis of published studies indicates that traces of antimicrobial agents are found both in raw materials and finished products [5,6]. Even concentrations that do not exceed established regulatory limits may exert long-term biological effects on the human body when regularly consumed with food [7].

Particular concern is associated with the impact of subtherapeutic doses of antibiotics on the emergence and spread of antimicrobial resistance (AMR). Products of animal origin may serve as reservoirs of resistant microorganisms and resistance genes formed under antibiotic pressure in livestock production [3,8]. Subsequently, such microorganisms may be transmitted to humans through the food chain and the environment.

Animal feed represents an additional and insufficiently studied source of antibiotic entry into the food chain [8,9,10]. Antibacterial agents may enter feed through intentional use, cross-contamination during production, storage, and transportation, as well as through plant-based components contaminated from the environment [9,10,11,12,13,14,15,16]. Even low concentrations of antibiotics in feed can affect the animal microbiota and create conditions favorable for the selection of resistant microorganisms [15,17,18].

Monitoring studies conducted in different countries demonstrate the heterogeneity of the situation. Antibiotics are most frequently detected in beef and pork, and less commonly in poultry meat, milk, and eggs [19,20]. At the same time, the limited range of analyzed compounds does not allow a comprehensive assessment of the actual scale of contamination. Recent scientific reviews emphasize that the issue of antibiotic residues extends beyond sanitary control alone and affects a broader range of challenges, including the sustainability of agri-food systems, public health protection, and the implementation of the One Health concept [8,21,22,23].

The presence of residual antibiotics in food products is directly associated with the global threat of AMR. According to the World Health Organization (WHO), approximately 700,000 people worldwide die annually from infections caused by resistant microorganisms, and by 2050, this number may reach 10 million [24,25,26,27]. Meat may serve as a reservoir of opportunistic microorganisms, including *Escherichia coli* and *Staphylococcus aureus*, which are characterized by high variability in resistance profiles [28,29,30,31,32,33].

A recent nationwide study in Kazakhstan demonstrated that antibiotic residues were present in all categories of meat and feed, with the highest levels detected in poultry meat and succulent feed [16]. However, that study focused exclusively on the quantitative analysis of residues and did not include the assessment of antimicrobial resistance in microorganisms.

The present study aimed to evaluate the presence of antibiotic residues in beef, pork, and animal feed from the Kostanay region, as well as to determine the antimicrobial resistance profiles of *E. coli* and *S. aureus* isolates recovered from meat samples.

## 2. Materials and Methods

### 2.1. Sample Collection and Study Objects

A cross-sectional study was conducted from September to December 2025 in farms of the Kostanay region engaged in cattle and pig breeding. Beef and pork samples were collected at licensed slaughterhouses, along with feed samples used in these farms. All sampling sites were under veterinary supervision and possessed the appropriate certificates and permits. In total, 29 beef samples, 25 pork samples, and 47 feed samples were collected. Feed samples were classified into three categories according to their composition and intended use: compound feed and premixes (*N* = 17), protein feed components (*N* = 15), and grain feed (*N* = 15). The analyzed feed materials included granules, premixes, compound feed, meal, oilcake, meat and bone meal, wheat, and barley.

Each sample was placed in a sterile plastic bag, labeled with a unique code, stored at a temperature of ≤4 °C, and transported to the laboratory within 24 h while maintaining the required cold chain conditions in accordance with ISO 17604:2015, as well as the current regulatory and legal requirements approved in the Republic of Kazakhstan, and ISO 6497:2002 for grain and feed additives [34,35].

### 2.2. Sample Preparation

The preparation of meat and feed samples for the determination of antibiotic residues using Randox Biochip Array Technology (Randox Laboratories Ltd., Crumlin, UK) included homogenization, buffer extraction, centrifugation, and collection of the supernatant for subsequent analysis. Samples were preliminarily homogenized to obtain a uniform consistency, after which the extraction of antimicrobial compounds was performed according to the manufacturer’s protocol. The obtained extracts were centrifuged, and the supernatant was used for further analysis using the biochip analyzer.

To improve measurement accuracy and ensure reproducibility of the results, all stages of sample preparation were carried out under controlled laboratory conditions. All samples were recorded in the laboratory logbook and assigned individual identification codes.

### 2.3. Determination of Antibiotic Residues

For the determination of antibiotic residues (tetracycline, streptomycin, enrofloxacin, ceftiofur, thiamphenicol, and tylosin) in the investigated meat and feed samples, the multiplex immunochemical Randox Biochip Array technology (Randox, Crumlin, UK) was used. Two validated analytical panels were applied: Antimicrobial Array Panel II Plus EV3524 A/B and Antimicrobial Array Panel II Plus EV4169 A/B. The panel enables simultaneous detection of multiple classes of antibiotic residues in a single sample. All analyses were performed according to the manufacturer’s instructions under standardized laboratory conditions. The limits of detection (LOD) depended on the analyzed matrix and antimicrobial compound in accordance with the manufacturer’s specifications. For feed samples, the LOD values were 10.00 µg/kg for quinolones, tetracyclines, and tylosin; 15.00 µg/kg for ceftiofur and thiamphenicol; and 80.00 µg/kg for streptomycin. For meat samples, the LOD values were 5.00 µg/kg for quinolones, 4.60 µg/kg for ceftiofur, 1.30 µg/kg for thiamphenicol, 7.00 µg/kg for streptomycin, 0.90 µg/kg for tylosin, and 4.80 µg/kg for tetracyclines. The maximum residue limits (MRLs) applied in the study were 0.1 mg/kg for tetracyclines, quinolones, and tylosin; 0.5 mg/kg for streptomycin; 1 mg/kg for ceftiofur; and 0.05 mg/kg for thiamphenicol.

Calibration was performed using a 9-point calibration curve. Calibrators were reconstituted in 1 mL of deionized water, vortex-mixed for 30 min, and used immediately. Each kit included quality control materials for method performance validation. All procedures were carried out in accordance with Randox validation protocols and the recommendations of the European Medicines Agency.

### 2.4. Microbiological Analysis

Beef and pork samples were examined for the presence of *Salmonella* spp., *Listeria monocytogenes*, *S. aureus*, *E. coli*, *Campylobacter* spp., and *Yersinia* spp.

Microbiological analyses were performed using standard culture-based methods for microorganism isolation in accordance with international ISO standards: detection of *Salmonella* spp. (ISO 6579-1:2017), *L. monocytogenes* (ISO 11290-1:2017), *Campylobacter* spp. (ISO 10272-1:2017), *S. aureus* (ISO 6888-1:2021), *E. coli* (ISO 16649-2:2001), and *Yersinia* spp. (ISO 10273:2017) [36,37,38,39,40,41].

Final confirmation of bacterial isolates was carried out using matrix-assisted laser desorption/ionization time-of-flight mass spectrometry (MALDI-TOF MS) with a MALDI Biotyper Sirius System IV and Bruker Real-Time Classification software version 5.1.450.324 (Bruker Daltonics GmbH & Co. KG, Bremen, Germany). Identification was performed by comparison of the obtained mass spectra with the reference database according to the manufacturer’s instructions. Score values in the range of 1.8–2.0 were considered indicative of reliable species-level identification [42].

### 2.5. Antimicrobial Susceptibility Testing

All confirmed isolates of *E. coli* and *S. aureus* obtained during microbiological examination of beef and pork samples were included in the antimicrobial susceptibility analysis. In total, 16 *E. coli* isolates and 9 *S. aureus* isolates were analyzed.

Antimicrobial susceptibility was determined using the Kirby-Bauer disk diffusion method on Mueller-Hinton agar with six antibiotic disks (NICF, LLC; St. Petersburg, Russia): cefoxitin (FOX)—30 µg, tetracycline (TET)—30 µg, enrofloxacin (ENR)—5 µg, streptomycin (STR)—10 µg, thiamphenicol (THI)—30 µg, and tylosin (TYL)—15 µg. The bacterial suspension was standardized to a density equivalent to 0.5 McFarland standard. After application of the antibiotic disks, the plates were incubated at 37 °C for 18–24 h. The results were evaluated by measuring the diameters of growth inhibition zones followed by interpretation according to the European Committee on Antimicrobial Susceptibility Testing (EUCAST) guidelines (version 15.0, 2024) [43].

### 2.6. Statistical Analysis

Statistical data analysis was performed using Microsoft Excel 2010 (Microsoft Corp., Redmond, WA, USA). Descriptive statistics for all studied parameters were calculated and presented as mean ± standard deviation (SD). One-way analysis of variance (ANOVA) was used to evaluate overall differences in antibiotic residue concentrations among feed groups. Differences were considered statistically significant at *p* < 0.05.

## 3. Results

### 3.1. Sampling Strategies

For meat sampling, a random sampling method was applied within the available product batches at slaughter points, which minimized selection bias and ensured sample representativeness within the framework of the cross-sectional study design. Feed samples, in contrast, were collected purposively from the diets used at the respective farms, taking into account the actual feeding structure of the animals.

### 3.2. Residual Levels of Antibiotics in Meat

The obtained results were evaluated with consideration of the maximum residue limits (MRLs) established by international and national regulatory documents, including the requirements of the Codex Alimentarius Commission and European Union regulations. The detected antibiotic concentrations were compared with the current regulatory limits established for food products of animal origin.

Table 1 presents the results of antibiotic residue determination in beef samples (*N* = 29). Residual amounts of antibiotics were detected in a portion of the analyzed samples, with all concentrations remaining below the established MRLs.

Tetracycline residues were detected in 22 beef samples (75.9%), with a mean concentration of 0.00762 mg/kg, which is well below the MRL (0.1 mg/kg). Streptomycin was identified in 23 samples (79.3%) with an average level of 0.02151 mg/kg, compared to the permissible limit of 0.5 mg/kg.

Quinolone residues were detected in 22 samples (75.9%) at a mean concentration of 0.00152 mg/kg. Ceftiofur and thiamphenicol were identified in 12 samples (41.4%), with concentrations of 0.00968 and 0.00253 mg/kg, respectively. Tylosin was detected in 13 samples (44.8%) with a mean level of 0.00176 mg/kg.

Similar results were obtained in the analysis of pork samples (*N* = 25) (Table 2).

Tetracycline residues were detected in 21 samples (84.0%) with a mean concentration of 0.00983 mg/kg. Streptomycin was identified in 22 samples (88.0%) with an average level of 0.05069 mg/kg.

Quinolone residues were detected in 21 samples (84.0%) at a concentration of 0.00182 mg/kg. Ceftiofur was identified in 16 samples (64.0%), thiamphenicol in 17 samples (68.0%), and tylosin in 19 samples (76.0%).

The concentrations of all studied antibiotics in pork samples were also significantly below the established MRLs.

Thus, antibiotic residues were detected in both beef and pork with varying frequencies; however, in all cases, their levels did not exceed the established regulatory limits.

### 3.3. Antibiotic Residues in Feed

Residual amounts of antibiotics were detected in 46 out of 47 analyzed feed samples (97.9%). In one sample (2.1%), the concentrations of all investigated antibiotics were below the limit of quantification.

All six antibiotics (tetracyclines, streptomycin, enrofloxacin, ceftiofur, thiamphenicol, and tylosin) were detected with equally high frequency, each being present in 46 samples (97.9%). Simultaneous presence of all six antibiotics was identified in 46 samples (97.9%), indicating systemic multi-contamination of feed raw materials.

Table 3 presents the results of the analysis of cattle and pig feed samples for antibiotic residues.

The analysis of compound feed, protein feed components, and grain feed samples revealed the presence of all studied antibiotics at trace levels in the mg/kg range. Their concentrations varied considerably depending on the type of feed.

The highest concentrations for all antibiotics were recorded in protein feed components. In particular, the level of ceftiofur reached 0.02316 mg/kg, Quinolones 0.00787 mg/kg, and tetracyclines 0.00835 mg/kg. A similar pattern was observed for streptomycin, thiamphenicol, and tylosin.

In compound feed, antibiotic levels were generally intermediate. Specifically, ceftiofur concentrations reached 0.01103 mg/kg, tetracyclines 0.00505 mg/kg, and enrofloxacin 0.00396 mg/kg.

The lowest concentrations of all studied antibiotics were detected in grain feed, where values ranged from 0.00107 to 0.00579 mg/kg, which is significantly lower compared to other types of feed.

Statistical analysis demonstrated significant differences between feed groups for all studied antibiotics (*p* < 0.001), indicating a systematic distribution pattern of residual amounts and excluding the possibility of random variation in the obtained data.

### 3.4. Prevalence of Microorganisms in Beef and Pork Samples

To assess the microbiological safety of beef and pork, the samples were examined for the following groups of microorganisms: *Salmonella* spp., *L. monocytogenes*, *S. aureus*, *E. coli*, *Campylobacter* spp., and *Yersinia* spp. According to the study results, only *S. aureus* and *E. coli* were detected in the meat samples; therefore, further analysis was focused on these microorganisms.

Microbiological analysis of beef (*N* = 29) and pork (*N* = 25) samples collected at slaughterhouses resulted in the isolation of bacteria belonging to the genera *E. coli* and *S. aureus* (Table 4).

*E. coli* was the most frequently detected microorganism, identified in 9 out of 29 beef samples (31%) and in 7 out of 25 pork samples (28%). *S. aureus* was detected less frequently, being present in 6 beef samples (20.6%) and 3 pork samples (12%).

Overall, the prevalence of microorganisms was moderate and comparable between beef and pork, with a slightly higher occurrence of *S. aureus* observed. The obtained results reflect the presence of typical microflora in raw meat and are likely associated with slaughtering and primary processing stages rather than indicating violations of sanitary requirements.

### 3.5. Antimicrobial Resistance of Microorganisms

Within the framework of the study, resistance analysis was performed for bacterial isolates of *E. coli* (*n* = 16) and *S. aureus* (*n* = 9) obtained from beef and pork samples. The results are presented in Table 5. Antimicrobial susceptibility profiles of all *E. coli* and *S. aureus* isolates were determined against six antimicrobial agents included in the Randox residue analysis panel. These agents were selected for their veterinary relevance and suitability for assessing the relationship between antibiotic residues and antimicrobial resistance.

*E. coli* isolates showed a moderate prevalence of resistance to tetracycline (44.4%) and streptomycin (44.4%), while resistance to enrofloxacin and tylosin was not detected. In contrast, *S. aureus* isolates demonstrated a high prevalence of resistance to streptomycin (83.3%) and moderate prevalence of resistance to cefoxitin, tetracycline, and thiamphenicol (33.3–50.0%).

**Figure 1:**
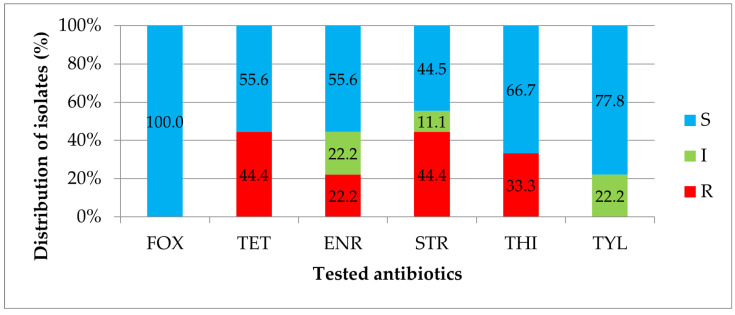
Antimicrobial susceptibility profiles of *E. coli* isolates from beef samples (%). Notes: S, susceptible; I, susceptible, increased exposure; R, resistant; FOX, cefoxitin; TET, tetracycline; ENR, enrofloxacin; STR, streptomycin; THI, thiamphenicol; TYL, tylosin.

The distribution of antimicrobial susceptibility among *E. coli* isolates from beef is shown in Figure 1. Most isolates were susceptible to the tested antibiotics, with complete susceptibility to cefoxitin (100%). The highest proportion of resistant isolates was observed for tetracycline and streptomycin (44.4%). Lower proportions of resistant isolates were detected for enrofloxacin (22.2%) and thiamphenicol (33.3%).

**Figure 2:**
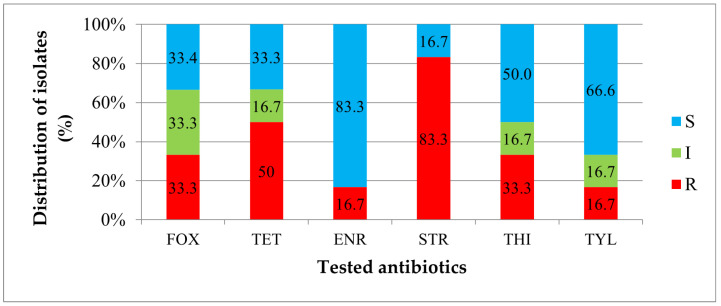
Antimicrobial susceptibility profiles of *S. aureus* isolates from beef samples (%). Notes: S, susceptible; I, susceptible, increased exposure; R, resistant; FOX, cefoxitin; TET, tetracycline; ENR, enrofloxacin; STR, streptomycin; THI, thiamphenicol; TYL, tylosin.

The antimicrobial susceptibility profile of *S. aureus* isolates from beef is presented in Figure 2. A high level of resistance was observed for streptomycin (83.3%) and tetracycline (50.0%), while moderate resistance was detected for cefoxitin and thiamphenicol (33.3%). Resistance to enrofloxacin and tylosin was lower (16.7%).

Overall, the observed resistance levels demonstrate the diversity of antimicrobial resistance profiles among microorganisms isolated from beef samples.

Antimicrobial resistance profiles of *E. coli* and *S. aureus* isolates obtained from pork samples are summarized in Table 6. High resistance rates to several antimicrobial agents were observed among pork isolates. *E. coli* isolates showed the highest resistance to tetracycline and thiamphenicol, while *S. aureus* isolates demonstrated complete resistance to thiamphenicol and high resistance to tetracycline, enrofloxacin, and streptomycin.

**Figure 3:**
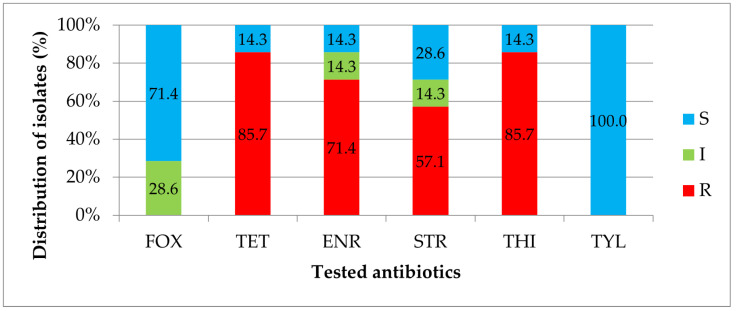
Antimicrobial susceptibility profiles of *E. coli* isolates from pork samples (%). Notes: S, susceptible; I, susceptible, increased exposure; R, resistant; FOX, cefoxitin; TET, tetracycline; ENR, enrofloxacin; STR, streptomycin; THI, thiamphenicol; TYL, tylosin.

For *E. coli* isolates from pork, the highest proportions of resistant isolates were observed for tetracycline (85.7%) and thiamphenicol (85.7%), followed by enrofloxacin (71.4%) and streptomycin (57.1%). In contrast, susceptibility to cefoxitin predominated (71.4%), while no resistance to tylosin was detected (Figure 3).

**Figure 4:**
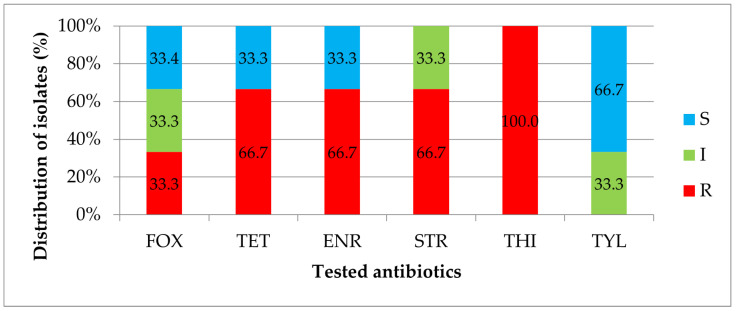
Antimicrobial susceptibility profiles of *S. aureus* isolates from pork samples (%). Notes: S, susceptible; I, susceptible, increased exposure; R, resistant; FOX, cefoxitin; TET, tetracycline; ENR, enrofloxacin; STR, streptomycin; THI, thiamphenicol; TYL, tylosin.

For *S. aureus* isolates from pork, complete resistance was observed to thiamphenicol (100%), while resistance to tetracycline, enrofloxacin, and streptomycin reached 66.7% for each antimicrobial. Resistance to cefoxitin was detected in 33.3% of isolates, which may suggest the presence of possible MRSA-associated resistance; however, this finding should be considered as a screening result requiring confirmatory testing. The highest susceptibility was observed to tylosin (66.7%) (Figure 4).

Overall, higher proportions of resistant isolates were observed in pork than in beef. Differences in susceptibility profiles between isolates from beef and pork were observed for several tested antimicrobials. The distribution of resistant, susceptible, and intermediate isolates varied depending on both the bacterial species and the type of meat sample.

## 4. Discussion

The MRLs for antibiotics in food products of animal origin applied in this study were based on the requirements of the technical regulations of the Eurasian Economic Union (EAEU) [44], including those adopted in the Republic of Kazakhstan, and are generally consistent with international standards such as the Codex Alimentarius and Commission Regulation (EU) No. 37/2010. MRLs established within the EAEU are comparable to, and in some cases more stringent than, those of the European Union [45,46,47].

In addition to the findings of the present study, updated requirements for the control of veterinary drug residues in products of animal origin entered into force in January 2026 in the countries of the Eurasian Economic Union, including Russia and Kazakhstan [48]. The amendments introduced MRLs for 75 veterinary medicinal substances and strengthened regulatory control over veterinary drug residues in food products of animal origin.

The results of the study demonstrated that antibiotic residues are present at trace levels in meat (beef and pork) produced in the Kostanay region and remain within established regulatory limits. Similar findings have been reported in a number of international studies, indicating the widespread occurrence of antibiotic residues in food products of animal origin even under compliance with regulatory requirements. In particular, annual reports of the European Food Safety Authority show that residues of veterinary medicinal products in food generally do not exceed established limits, yet are regularly detected during monitoring activities [49]. Furthermore, studies by Ghimpețeanu et al. confirm that, despite regulatory compliance, low-level contamination of animal-derived food products with antibiotics is widespread globally due to their use in livestock production, posing potential risks to public health [8].

It should be noted that, within the framework of the present study, trace levels of antibiotic residues were also detected in feed (compound feed, protein feed components, and grain feed), indicating a possible route of their entry into the animal organism and subsequent accumulation in products of animal origin. In the studied farms, the main proportion of the diet consisted of locally produced feed, reflecting the agricultural characteristics of the region. At the same time, commercial feed additives were included in the rations at a level of 10–30%, the use of which may contribute to the introduction of trace amounts of antibiotics. The presence of antibiotic residues in the feed base may be associated not only with the use of commercial additives but also with environmental factors, including water, plants, and organic fertilizers (manure). This may be linked to previously uncontrolled use of antimicrobial agents in livestock production, contributing to their accumulation in the environment.

Studies conducted in several countries have shown that antibiotics may be present in feed components and circulate within the “feed–animal–environment” system [50]. According to Zhang et al., the presence of antibiotics in feed may be determined by technological features of production, including cross-contamination [51]. In addition, Sarmah et al. reported that antimicrobial substances can persist in the environment and enter feed through soil and plants, forming additional pathways for their incorporation into the food chain [52].

In contrast to food products of animal origin, MRLs for antibiotics are generally not established for feed and compound feed. In the European Union and the EAEU, regulatory control is primarily focused on the proper use of veterinary medicinal products and the prevention of cross-contamination, while safety is assessed based on residue levels in food products of animal origin.

At the same time, the detection of antibiotics directly in feed, including when commercial additives are incorporated into rations, allows feed to be considered as one of the probable sources of these substances entering the animal organism.

Thus, the obtained results indicate the complex nature of contamination, which requires further research to clarify the contribution of various factors and pathways of antibiotic entry into the “feed–animal–product” system.

It is important to note that even in the absence of exceedances of MRLs in most samples, the presence of antibiotic residues in food products is considered a potential risk factor for public health. Ghimpețeanu et al. emphasize that compliance with withdrawal periods and established MRLs does not guarantee complete product safety, as low concentrations of antibiotics may induce toxic and immunopathological effects and contribute to the development of antimicrobial resistance [8]. Similar conclusions are reported by Sadighara et al., who demonstrated that minimal concentrations of antibiotics in food products can reduce the diversity of the intestinal microbiota and alter microbial metabolic pathways, potentially decreasing the effectiveness of antibacterial therapy under chronic exposure [24].

In addition to various adverse health effects associated with exposure to antibiotic residues, one of the key concerns is the development of antimicrobial resistance in microorganisms. The regular intake of subtherapeutic doses of antimicrobial agents through food may exert selective pressure on the microbiota, potentially contributing to the selection and circulation of resistant strains and reduced effectiveness of antibacterial therapy. Similar conclusions have been presented in several international studies, highlighting the role of low antibiotic concentrations in the development of microbial resistance [26,53,54].

In this context, particular importance is attached to the results obtained from the analysis of antimicrobial resistance in microorganisms isolated from meat products [55]. In the present study, isolates of *E. coli* and *S. aureus* demonstrated varying degrees of resistance to several classes of antibiotics, including tetracyclines, fluoroquinolones, aminoglycosides, and phenicols. Similar findings have been reported by Marshall and Levy, who showed that the use of antimicrobial agents in livestock production is associated with the emergence of resistant bacterial strains capable of transmission through the food chain [56,57].

At the same time, higher levels of antimicrobial resistance were identified in isolates obtained from pork compared to beef, which is consistent with studies reporting more intensive antimicrobial use in pig production systems. Particular attention should be paid to the detection of cefoxitin-resistant *S. aureus* strains, which may indicate the presence of methicillin-resistant forms (MRSA). However, resistance to cefoxitin is considered a screening marker for MRSA and requires confirmation by additional phenotypic or molecular genetic methods. Similar isolates were previously described by Smith et al. [58].

Comparison of the obtained data with the results of antibiotic residue analysis in feed and meat products demonstrated that the identified resistance profiles were consistent with the detection of trace amounts of the corresponding antimicrobial agents in the studied samples. Even low concentrations of antibiotics may contribute to selective pressure on the microbiota and support the circulation of resistant microorganisms, which is consistent with current concepts regarding the role of sub-inhibitory concentrations of antimicrobial agents in the development of antimicrobial resistance [16,59,60,61].

The results obtained complement existing data on the prevalence of antimicrobial residues and antimicrobial resistance in microorganisms.

Thus, the findings of the present study are consistent with those reported by international authors and confirm that the issue of antibiotic residues in products of animal origin remains relevant even under regulatory control of their use. The detection of trace concentrations of antimicrobial substances in food products entering the market supports the need for systematic monitoring focused not only on controlling exceedances of MRLs but also on assessing potential long-term risks, including the development of antimicrobial resistance.

Despite the obtained results, several limitations of the present study should be considered. The study was limited to one region of Kazakhstan and included a relatively moderate number of meat and feed samples. In addition, molecular confirmation of antimicrobial resistance genes was not performed, and antimicrobial resistance analysis was limited to bacterial isolates obtained from meat samples. Further large-scale studies involving molecular methods and broader geographic coverage are required to improve the understanding of the relationship between antibiotic residues and antimicrobial resistance in the food chain.

## 5. Conclusions

The present study demonstrated that beef and pork samples produced in the Kostanay region contain antibiotic residues at trace levels that do not exceed established MRLs. At the same time, the presence of antimicrobial substances was identified in feed, including compound feed, protein components, and grain, indicating possible routes of their entry into the animal organism.

Microbiological analysis confirmed the presence of opportunistic microorganisms, among which isolates of *E. coli* and *S. aureus* exhibited varying degrees of resistance to the studied antibiotics. A tendency toward higher resistance was observed in isolates obtained from pork, and cefoxitin-resistant *S. aureus* strains were identified, indicating the presence of methicillin-resistant forms.

The obtained data indicate the presence of even trace concentrations of antibiotics together with resistant bacterial isolates in the studied samples, highlighting the need for further improvement of monitoring systems aimed at the comprehensive assessment of contamination in feed and products of animal origin, as well as the associated risks of antimicrobial resistance development.

## Figures and Tables

**Table 1:** Antibiotic residues in beef samples.

Antimicrobial Agent	*N*	*n*	Mean ± SD (mg/kg)	Range (mg/kg)
Tetracyclines	29	22	0.00762 ± 0.00367	0.00181–0.01240
Streptomycin	29	23	0.02151 ± 0.02264	0.00245–0.09609
Quinolones	29	22	0.00152 ± 0.00115	0.00010–0.00322
Ceftiofur	29	12	0.00968 ± 0.00424	0.00306–0.01613
Thiamphenicol	29	12	0.00253 ± 0.00124	0.00072–0.00483
Tylosin	29	13	0.00176 ± 0.00070	0.00011–0.00250

Notes: *N*—total number of examined samples; *n*—number of positive samples; SD—standard deviation.

**Table 2:** Antibiotic residues in pork samples.

Antimicrobial Agent	*N*	*n*	Mean ± SD (mg/kg)	Range (mg/kg)
Tetracyclines	25	21	0.00983 ± 0.00354	0.00479–0.01611
Streptomycin	25	22	0.05069 ± 0.04348	0.00201–0.17175
Quinolones	25	21	0.00182 ± 0.00098	0.00027–0.00308
Ceftiofur	25	16	0.00852 ± 0.00456	0.00157–0.01436
Thiamphenicol	25	17	0.00212 ± 0.00135	0.00018–0.00473
Tylosin	25	19	0.00149 ± 0.00072	0.00019–0.00242

Notes: *N*—total number of examined samples; *n*—number of positive samples; SD—standard deviation.

**Table 3:** Antibiotic residues in feed.

Antimicrobial Agent	Compound Feed and Premixes ± SD (mg/kg) (*N* = 17)	Protein Feed Components ± SD (mg/kg) (*N* = 15)	Grain Feed ± SD (mg/kg) (*N* = 15)
Tetracyclines	0.00505 ± 0.00223	0.00835 ± 0.00122	0.00475 ± 0.00028
Streptomycin	0.00428 ± 0.00098	0.00515 ± 0.00046	0.00292 ± 0.00045
Quinolones	0.00396 ± 0.00159	0.00787 ± 0.00507	0.00205 ± 0.00043
Ceftiofur	0.01103 ± 0.00401	0.02316 ± 0.01068	0.00579 ± 0.00067
Thiamphenicol	0.00300 ± 0.00103	0.00484 ± 0.00208	0.00107 ± 0.00022
Tylosin	0.00361 ± 0.00101	0.00412 ± 0.00088	0.00149 ± 0.00020

Notes: *N*—total number of examined samples; SD—standard deviation.

**Table 4:** Prevalence of detected microorganisms in beef and pork samples.

Microorganism	Beef (*n*/*N*, %)	Pork (*n*/*N*, %)
*E. coli*	9/29 (31%)	7/25 (28%)
*S. aureus*	6/29 (20.6%)	3/25 (12%)

Notes: *n*—number of positive samples; *N*—total number of examined samples; %—percentage of positive samples.

**Table 5:** Antimicrobial resistance profiles of microorganisms isolated from beef.

Microorganism	*n*	FOX (%)	TET (%)	ENR (%)	STR (%)	THI (%)	TYL (%)
*E. coli*	9	-	44.4	22.2	44.4	33.3	-
*S. aureus*	6	33.3	50.0	16.7	83.3	33.3	16.7

Notes: *n*, number of isolates; FOX, cefoxitin; TET, tetracycline; ENR, enrofloxacin; STR, streptomycin; THI, thiamphenicol; TYL, tylosin; “-”, indicates absence of resistant isolates.

**Table 6:** Antimicrobial resistance profiles of microorganisms isolated from pork.

Microorganism	*n*	FOX (%)	TET (%)	ENR (%)	STR (%)	THI (%)	TYL (%)
*E. coli*	7	-	85.7	71.4	57.1	85.7	-
*S. aureus*	3	33.3	66.7	66.7	66.7	100	-

Notes: *n*, number of isolates; FOX, cefoxitin; TET, tetracycline; ENR, enrofloxacin; STR, streptomycin; THI, thiamphenicol; TYL, tylosin; “-”, indicates absence of resistant isolates.

## Data Availability

The original contributions presented in the study are included in the article, further inquiries can be directed to the corresponding authors.
